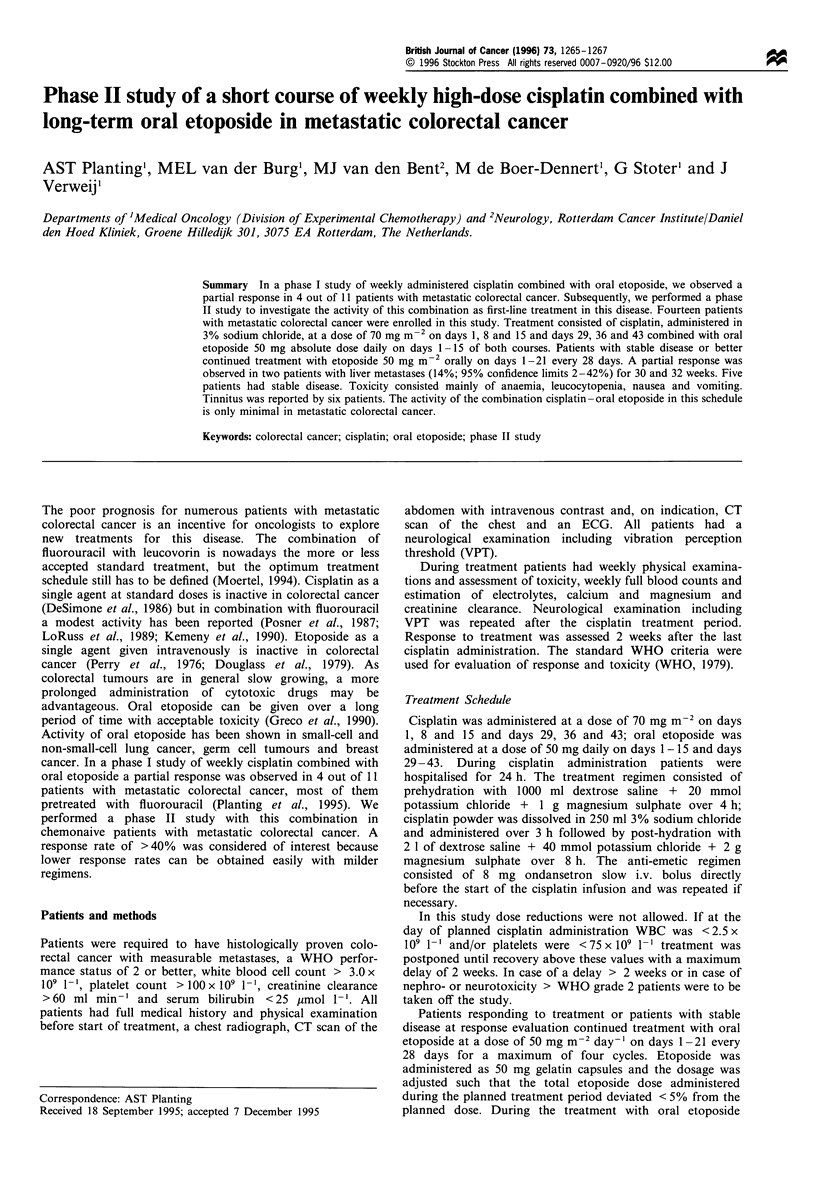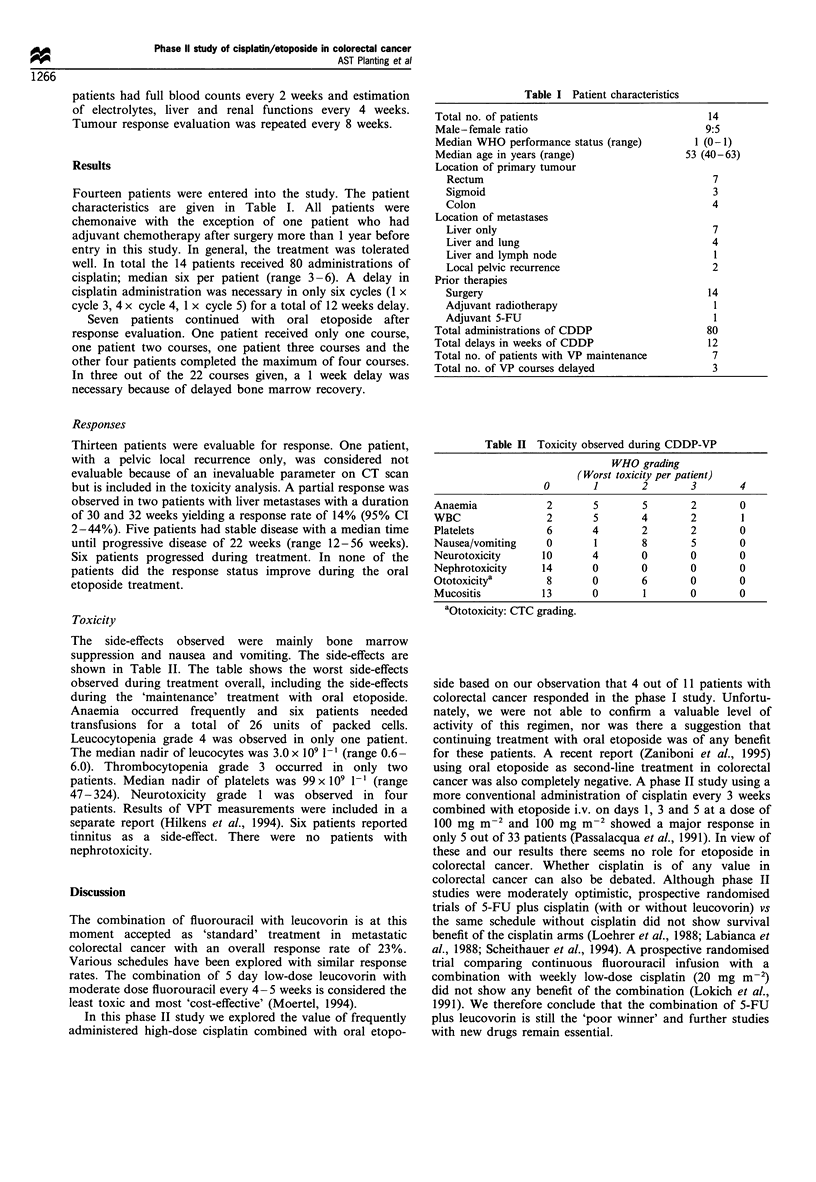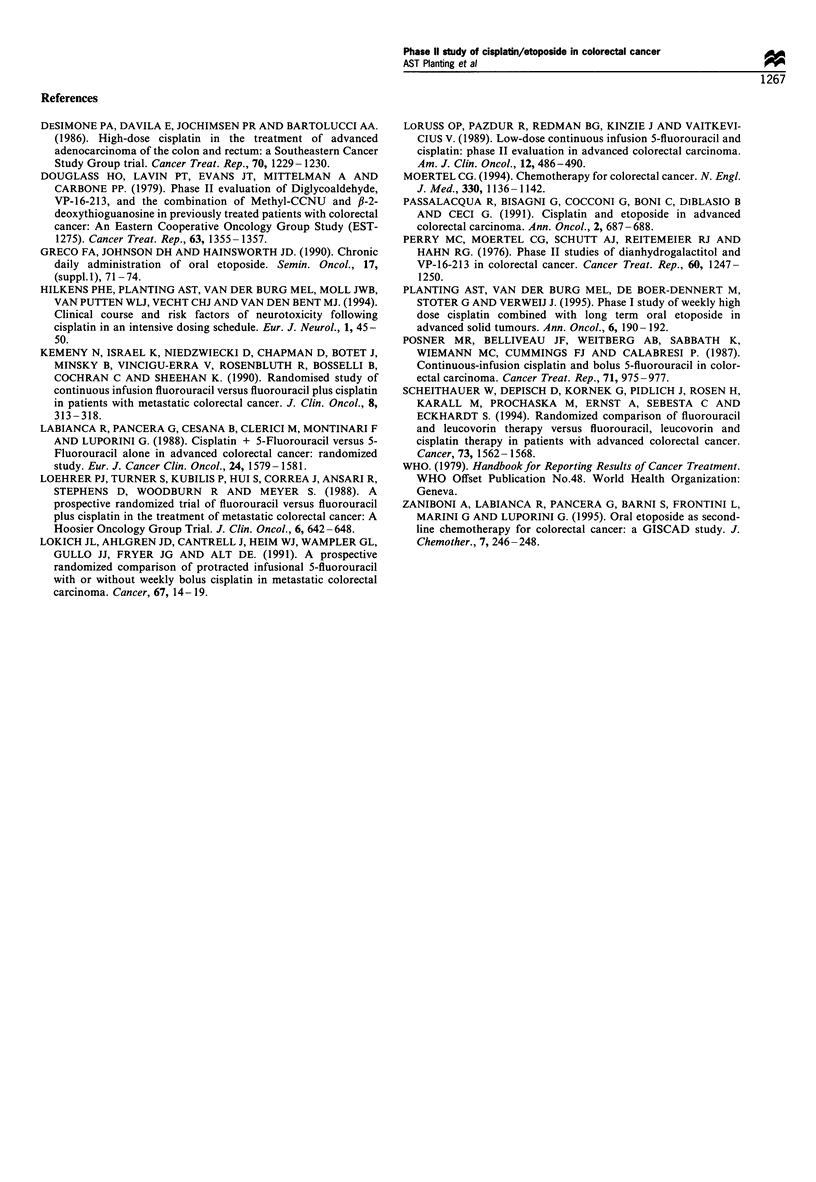# Phase II study of a short course of weekly high-dose cisplatin combined with long-term oral etoposide in metastatic colorectal cancer.

**DOI:** 10.1038/bjc.1996.242

**Published:** 1996-05

**Authors:** A. S. Planting, M. E. van der Burg, M. J. van den Bent, M. de Boer-Dennert, G. Stoter, J. Verweij

**Affiliations:** Department of Medical Oncology (Division of Experimental Chemotherapy), Rotterdam Cancer Institute, The Netherlands.

## Abstract

In a phase I study of weekly administered cisplatin combined with oral etoposide, we observed a partial response in 4 out of 11 patients with metastatic colorectal cancer. Subsequently, we performed a phase II study to investigate the activity of this combination as first-line treatment in this disease. Fourteen patients with metastatic colorectal cancer were enrolled in this study. Treatment consisted of cisplatin, administered in 3% sodium chloride, at a dose of 70 mg m-2 on days 1, 8 and 15 and days 29, 36 and 43 combined with oral etoposide 50 mg absolute dose daily on days 1-15 of both courses. Patients with stable disease or better continued treatment with etoposide 50 mg m-2 orally on days 1-21 every 28 days. A partial response was observed in two patients with liver metastases (14%; 95% confidence limits 2-42%) for 30 and 32 weeks. Five patients had stable disease. Toxicity consisted mainly of anaemia, leucocytopenia, nausea and vomiting. Tinnitus was reported by six patients. The activity of the combination cisplatin-oral etoposide in the schedule is only minimal in metastatic colorectal cancer.


**British Journal of Cancer (1996) 73,** 1265-1267
© 1996 Stockton Press All rights reserved 0007-0920/96 $12.00

### Phase II study of a short course of weekly high-dose cisplatin combined with long-term oral etoposide in metastatic colorectal cancer

AST Planting[1], MEL van der Burg[1], MJ van den Bent[2], M de Boer-Dennert[1], G Stoter[1] and J Verweij[1]

*Departments of [1]Medical Oncology (Division of Experimental Chemotherapy) and [2]Neurology, Rotterdam Cancer Institute/Daniel den Hoed Kliniek, Groene Hilledijk 301, 3075 EA Rotterdam, The Netherlands.*

**Summary** In a phase I study of weekly administered cisplatin combined with oral etoposide, we observed a partial response in 4 out of 11 patients with metastatic colorectal cancer. Subsequently, we performed a phase II study to investigate the activity of this combination as first-line treatment in this disease. Fourteen patients with metastatic colorectal cancer were enrolled in this study. Treatment consisted of cisplatin, administered in 3% sodium chloride, at a dose of 70 mg $m^{-2}$ on days 1, 8 and 15 and days 29, 36 and 43 combined with oral etoposide 50 mg absolute dose daily on days 1-15 of both courses. Patients with stable disease or better continued treatment with etoposide 50 mg $m^{-2}$ orally on days 1-21 every 28 days. A partial response was observed in two patients with liver metastases (14%; 95% confidence limits 2-42%) for 30 and 32 weeks. Five patients had stable disease. Toxicity consisted mainly of anaemia, leucocytopenia, nausea and vomiting. Tinnitus was reported by six patients. The activity of the combination cisplatin-oral etoposide in this schedule is only minimal in metastatic colorectal cancer.

**Keywords:** colorectal cancer; cisplatin; oral etoposide; phase II study

The poor prognosis for numerous patients with metastatic colorectal cancer is an incentive for oncologists to explore new treatments for this disease. The combination of fluorouracil with leucovorin is nowadays the more or less accepted standard treatment, but the optimum treatment schedule still has to be defined (Moertel, 1994). Cisplatin as a single agent at standard doses is inactive in colorectal cancer (DeSimone *et al.*, 1986) but in combination with fluorouracil a modest activity has been reported (Posner *et al.*, 1987; LoRuss *et al.*, 1989; Kemeny *et al.*, 1990). Etoposide as a single agent given intravenously is inactive in colorectal cancer (Perry *et al.*, 1976; Douglass *et al.*, 1979). As colorectal tumours are in general slow growing, a more prolonged administration of cytotoxic drugs may be advantageous. Oral etoposide can be given over a long period of time with acceptable toxicity (Greco *et al.*, 1990). Activity of oral etoposide has been shown in small-cell and non-small-cell lung cancer, germ cell tumours and breast cancer. In a phase I study of weekly cisplatin combined with oral etoposide a partial response was observed in 4 out of 11 patients with metastatic colorectal cancer, most of them pretreated with fluorouracil (Planting *et al.*, 1995). We performed a phase II study with this combination in chemonaive patients with metastatic colorectal cancer. A response rate of >40% was considered of interest because lower response rates can be obtained easily with milder regimens.

### Patients and methods

Patients were required to have histologically proven colorectal cancer with measurable metastases, a WHO performance status of 2 or better, white blood cell count > $3.0 \times 10^9$ $l^{-1}$, platelet count $>100 \times 10^9$ $l^{-1}$, creatinine clearance >60 ml $min^{-1}$ and serum bilirubin <25 $\mu$mol $l^{-1}$. All patients had full medical history and physical examination before start of treatment, a chest radiograph, CT scan of the abdomen with intravenous contrast and, on indication, CT scan of the chest and an ECG. All patients had a neurological examination including vibration perception threshold (VPT).

During treatment patients had weekly physical examinations and assessment of toxicity, weekly full blood counts and estimation of electrolytes, calcium and magnesium and creatinine clearance. Neurological examination including VPT was repeated after the cisplatin treatment period. Response to treatment was assessed 2 weeks after the last cisplatin administration. The standard WHO criteria were used for evaluation of response and toxicity (WHO, 1979).

### *Treatment Schedule*

Cisplatin was administered at a dose of 70 mg $m^{-2}$ on days 1, 8 and 15 and days 29, 36 and 43; oral etoposide was administered at a dose of 50 mg daily on days 1-15 and days 29-43. During cisplatin administration patients were hospitalised for 24 h. The treatment regimen consisted of prehydration with 1000 ml dextrose saline + 20 mmol potassium chloride + 1 g magnesium sulphate over 4 h; cisplatin powder was dissolved in 250 ml 3% sodium chloride and administered over 3 h followed by post-hydration with 2 l of dextrose saline + 40 mmol potassium chloride + 2 g magnesium sulphate over 8 h. The anti-emetic regimen consisted of 8 mg ondansetron slow i.v. bolus directly before the start of the cisplatin infusion and was repeated if necessary.

In this study dose reductions were not allowed. If at the day of planned cisplatin administration WBC was $<2.5 \times 10^9$ $l^{-1}$ and/or platelets were $<75 \times 10^9$ $l^{-1}$ treatment was postponed until recovery above these values with a maximum delay of 2 weeks. In case of a delay > 2 weeks or in case of nephro- or neurotoxicity > WHO grade 2 patients were to be taken off the study.

Patients responding to treatment or patients with stable disease at response evaluation continued treatment with oral etoposide at a dose of 50 mg $m^{-2}$ $day^{-1}$ on days 1-21 every 28 days for a maximum of four cycles. Etoposide was administered as 50 mg gelatin capsules and the dosage was adjusted such that the total etoposide dose administered during the planned treatment period deviated <5% from the planned dose. During the treatment with oral etoposide

Correspondence: AST Planting
Received 18 September 1995; accepted 7 December 1995

patients had full blood counts every 2 weeks and estimation of electrolytes, liver and renal functions every 4 weeks. Tumour response evaluation was repeated every 8 weeks.

### Results

Fourteen patients were entered into the study. The patient characteristics are given in Table I. All patients were chemonaive with the exception of one patient who had adjuvant chemotherapy after surgery more than 1 year before entry in this study. In general, the treatment was tolerated well. In total the 14 patients received 80 administrations of cisplatin; median six per patient (range 3–6). A delay in cisplatin administration was necessary in only six cycles (1 × cycle 3, 4 × cycle 4, 1 × cycle 5) for a total of 12 weeks delay.

Seven patients continued with oral etoposide after response evaluation. One patient received only one course, one patient two courses, one patient three courses and the other four patients completed the maximum of four courses. In three out of the 22 courses given, a 1 week delay was necessary because of delayed bone marrow recovery.

**Table I** Patient characteristics

| | |
|---|---|
| Total no. of patients | 14 |
| Male–female ratio | 9:5 |
| Median WHO performance status (range) | 1 (0–1) |
| Median age in years (range) | 53 (40–63) |
| Location of primary tumour | |
| Rectum | 7 |
| Sigmoid | 3 |
| Colon | 4 |
| Location of metastases | |
| Liver only | 7 |
| Liver and lung | 4 |
| Liver and lymph node | 1 |
| Local pelvic recurrence | 2 |
| Prior therapies | |
| Surgery | 14 |
| Adjuvant radiotherapy | 1 |
| Adjuvant 5-FU | 1 |
| Total administrations of CDDP | 80 |
| Total delays in weeks of CDDP | 12 |
| Total no. of patients with VP maintenance | 7 |
| Total no. of VP courses delayed | 3 |

### *Responses*

Thirteen patients were evaluable for response. One patient, with a pelvic local recurrence only, was considered not evaluable because of an inevaluable parameter on CT scan but is included in the toxicity analysis. A partial response was observed in two patients with liver metastases with a duration of 30 and 32 weeks yielding a response rate of 14% (95% CI 2–44%). Five patients had stable disease with a median time until progressive disease of 22 weeks (range 12–56 weeks). Six patients progressed during treatment. In none of the patients did the response status improve during the oral etoposide treatment.

**Table II** Toxicity observed during CDDP-VP

| | *WHO grading (Worst toxicity per patient)* 0 | 1 | 2 | 3 | 4 |
|---|---|---|---|---|---|
| Anaemia | 2 | 5 | 5 | 2 | 0 |
| WBC | 2 | 5 | 4 | 2 | 1 |
| Platelets | 6 | 4 | 2 | 2 | 0 |
| Nausea/vomiting | 0 | 1 | 8 | 5 | 0 |
| Neurotoxicity | 10 | 4 | 0 | 0 | 0 |
| Nephrotoxicity | 14 | 0 | 0 | 0 | 0 |
| Ototoxicity[a] | 8 | 0 | 6 | 0 | 0 |
| Mucositis | 13 | 0 | 1 | 0 | 0 |

[a]Ototoxicity: CTC grading.

### *Toxicity*

The side-effects observed were mainly bone marrow suppression and nausea and vomiting. The side-effects are shown in Table II. The table shows the worst side-effects observed during treatment overall, including the side-effects during the 'maintenance' treatment with oral etoposide. Anaemia occurred frequently and six patients needed transfusions for a total of 26 units of packed cells. Leucocytopenia grade 4 was observed in only one patient. The median nadir of leucocytes was $3.0 \times 10^9\ l^{-1}$ (range 0.6–6.0). Thrombocytopenia grade 3 occurred in only two patients. Median nadir of platelets was $99 \times 10^9\ l^{-1}$ (range 47–324). Neurotoxicity grade 1 was observed in four patients. Results of VPT measurements were included in a separate report (Hilkens *et al.*, 1994). Six patients reported tinnitus as a side-effect. There were no patients with nephrotoxicity.

### Discussion

The combination of fluorouracil with leucovorin is at this moment accepted as 'standard' treatment in metastatic colorectal cancer with an overall response rate of 23%. Various schedules have been explored with similar response rates. The combination of 5 day low-dose leucovorin with moderate dose fluorouracil every 4–5 weeks is considered the least toxic and most 'cost-effective' (Moertel, 1994).

In this phase II study we explored the value of frequently administered high-dose cisplatin combined with oral etoposide based on our observation that 4 out of 11 patients with colorectal cancer responded in the phase I study. Unfortunately, we were not able to confirm a valuable level of activity of this regimen, nor was there a suggestion that continuing treatment with oral etoposide was of any benefit for these patients. A recent report (Zaniboni *et al.*, 1995) using oral etoposide as second-line treatment in colorectal cancer was also completely negative. A phase II study using a more conventional administration of cisplatin every 3 weeks combined with etoposide i.v. on days 1, 3 and 5 at a dose of 100 mg $m^{-2}$ and 100 mg $m^{-2}$ showed a major response in only 5 out of 33 patients (Passalacqua *et al.*, 1991). In view of these and our results there seems no role for etoposide in colorectal cancer. Whether cisplatin is of any value in colorectal cancer can also be debated. Although phase II studies were moderately optimistic, prospective randomised trials of 5-FU plus cisplatin (with or without leucovorin) *vs* the same schedule without cisplatin did not show survival benefit of the cisplatin arms (Loehrer *et al.*, 1988; Labianca *et al.*, 1988; Scheithauer *et al.*, 1994). A prospective randomised trial comparing continuous fluorouracil infusion with a combination with weekly low-dose cisplatin (20 mg $m^{-2}$) did not show any benefit of the combination (Lokich *et al.*, 1991). We therefore conclude that the combination of 5-FU plus leucovorin is still the 'poor winner' and further studies with new drugs remain essential.

### References

DESIMONE PA, DAVILA E, JOCHIMSEN PR AND BARTOLUCCI AA. (1986). High-dose cisplatin in the treatment of advanced adenocarcinoma of the colon and rectum: a Southeastern Cancer Study Group trial. *Cancer Treat. Rep.*, **70**, 1229–1230.

DOUGLASS HO, LAVIN PT, EVANS JT, MITTELMAN A AND CARBONE PP. (1979). Phase II evaluation of Diglycoaldehyde, VP-16-213, and the combination of Methyl-CCNU and $\beta$-2-deoxythioguanosine in previously treated patients with colorectal cancer: An Eastern Cooperative Oncology Group Study (EST-1275). *Cancer Treat. Rep.*, **63**, 1355–1357.

GRECO FA, JOHNSON DH AND HAINSWORTH JD. (1990). Chronic daily administration of oral etoposide. *Semin. Oncol.*, **17**, (suppl.1), 71–74.

HILKENS PHE, PLANTING AST, VAN DER BURG MEL, MOLL JWB, VAN PUTTEN WLJ, VECHT CHJ AND VAN DEN BENT MJ. (1994). Clinical course and risk factors of neurotoxicity following cisplatin in an intensive dosing schedule. *Eur. J. Neurol.*, **1**, 45–50.

KEMENY N, ISRAEL K, NIEDZWIECKI D, CHAPMAN D, BOTET J, MINSKY B, VINCIGU-ERRA V, ROSENBLUTH R, BOSSELLI B, COCHRAN C AND SHEEHAN K. (1990). Randomised study of continuous infusion fluorouracil versus fluorouracil plus cisplatin in patients with metastatic colorectal cancer. *J. Clin. Oncol.*, **8**, 313–318.

LABIANCA R, PANCERA G, CESANA B, CLERICI M, MONTINARI F AND LUPORINI G. (1988). Cisplatin + 5-Fluorouracil versus 5-Fluorouracil alone in advanced colorectal cancer: randomized study. *Eur. J. Cancer Clin. Oncol.*, **24**, 1579–1581.

LOEHRER PJ, TURNER S, KUBILIS P, HUI S, CORREA J, ANSARI R, STEPHENS D, WOODBURN R AND MEYER S. (1988). A prospective randomized trial of fluorouracil versus fluorouracil plus cisplatin in the treatment of metastatic colorectal cancer: A Hoosier Oncology Group Trial. *J. Clin. Oncol.*, **6**, 642–648.

LOKICH JL, AHLGREN JD, CANTRELL J, HEIM WJ, WAMPLER GL, GULLO JJ, FRYER JG AND ALT DE. (1991). A prospective randomized comparison of protracted infusional 5-fluorouracil with or without weekly bolus cisplatin in metastatic colorectal carcinoma. *Cancer*, **67**, 14–19.

LORUSS OP, PAZDUR R, REDMAN BG, KINZIE J AND VAITKEVICIUS V. (1989). Low-dose continuous infusion 5-fluorouracil and cisplatin: phase II evaluation in advanced colorectal carcinoma. *Am. J. Clin. Oncol.*, **12**, 486–490.

MOERTEL CG. (1994). Chemotherapy for colorectal cancer. *N. Engl. J. Med.*, **330**, 1136–1142.

PASSALACQUA R, BISAGNI G, COCCONI G, BONI C, DIBLASIO B AND CECI G. (1991). Cisplatin and etoposide in advanced colorectal carcinoma. *Ann. Oncol.*, **2**, 687–688.

PERRY MC, MOERTEL CG, SCHUTT AJ, REITEMEIER RJ AND HAHN RG. (1976). Phase II studies of dianhydrogalactitol and VP-16-213 in colorectal cancer. *Cancer Treat. Rep.*, **60**, 1247–1250.

PLANTING AST, VAN DER BURG MEL, DE BOER-DENNERT M, STOTER G AND VERWEIJ J. (1995). Phase I study of weekly high dose cisplatin combined with long term oral etoposide in advanced solid tumours. *Ann. Oncol.*, **6**, 190–192.

POSNER MR, BELLIVEAU JF, WEITBERG AB, SABBATH K, WIEMANN MC, CUMMINGS FJ AND CALABRESI P. (1987). Continuous-infusion cisplatin and bolus 5-fluorouracil in colorectal carcinoma. *Cancer Treat. Rep.*, **71**, 975–977.

SCHEITHAUER W, DEPISCH D, KORNEK G, PIDLICH J, ROSEN H, KARALL M, PROCHASKA M, ERNST A, SEBESTA C AND ECKHARDT S. (1994). Randomized comparison of fluorouracil and leucovorin therapy versus fluorouracil, leucovorin and cisplatin therapy in patients with advanced colorectal cancer. *Cancer*, **73**, 1562–1568.

WHO. (1979). *Handbook for Reporting Results of Cancer Treatment.* WHO Offset Publication No.48. World Health Organization: Geneva.

ZANIBONI A, LABIANCA R, PANCERA G, BARNI S, FRONTINI L, MARINI G AND LUPORINI G. (1995). Oral etoposide as second-line chemotherapy for colorectal cancer: a GISCAD study. *J. Chemother.*, **7**, 246–248.